# Predicting and Responding to Clinical Deterioration in Hospitalized Patients by Using Artificial Intelligence: Protocol for a Mixed Methods, Stepped Wedge Study

**DOI:** 10.2196/27532

**Published:** 2021-07-07

**Authors:** Laura M Holdsworth, Samantha M R Kling, Margaret Smith, Nadia Safaeinili, Lisa Shieh, Stacie Vilendrer, Donn W Garvert, Marcy Winget, Steven M Asch, Ron C Li

**Affiliations:** 1 Department of Medicine School of Medicine Stanford University Stanford, CA United States; 2 Center for Innovation to Implementation VA Palo Alto, CA United States

**Keywords:** artificial intelligence, clinical deterioration, rapid response team, mixed methods, workflow, predictive models, SEIPS 2.0

## Abstract

**Background:**

The early identification of clinical deterioration in patients in hospital units can decrease mortality rates and improve other patient outcomes; yet, this remains a challenge in busy hospital settings. Artificial intelligence (AI), in the form of predictive models, is increasingly being explored for its potential to assist clinicians in predicting clinical deterioration.

**Objective:**

Using the Systems Engineering Initiative for Patient Safety (SEIPS) 2.0 model, this study aims to assess whether an AI-enabled work system improves clinical outcomes, describe how the clinical deterioration index (CDI) predictive model and associated work processes are implemented, and define the emergent properties of the AI-enabled work system that mediate the observed clinical outcomes.

**Methods:**

This study will use a mixed methods approach that is informed by the SEIPS 2.0 model to assess both processes and outcomes and focus on how physician-nurse clinical teams are affected by the presence of AI. The intervention will be implemented in hospital medicine units based on a modified stepped wedge design featuring three stages over 11 months—stage 0 represents a baseline period 10 months before the implementation of the intervention; stage 1 introduces the CDI predictions to physicians only and triggers a physician-driven workflow; and stage 2 introduces the CDI predictions to the multidisciplinary team, which includes physicians and nurses, and triggers a nurse-driven workflow. Quantitative data will be collected from the electronic health record for the clinical processes and outcomes. Interviews will be conducted with members of the multidisciplinary team to understand how the intervention changes the existing work system and processes. The SEIPS 2.0 model will provide an analytic framework for a mixed methods analysis.

**Results:**

A pilot period for the study began in December 2020, and the results are expected in mid-2022.

**Conclusions:**

This protocol paper proposes an approach to evaluation that recognizes the importance of assessing both processes and outcomes to understand how a multifaceted AI-enabled intervention affects the complex team-based work of identifying and managing clinical deterioration.

**International Registered Report Identifier (IRRID):**

PRR1-10.2196/27532

## Introduction

### Background

The timely identification of hospitalized patients who are clinically deteriorating is critical for facilitating prompt clinical interventions to improve patient outcomes but remains challenging for hospital systems to perform consistently. Artificial intelligence (AI), in the form of statistical models that predict clinical deterioration, is increasingly being considered by hospitals to aid in the early identification of these patients. Many such prediction models have been developed and reported in the literature, ranging from the sequential organ failure assessment score, which predicts inpatient mortality [1], to more recently developed machine learning models that predict a variety of outcomes, such as transfer to the intensive care unit (ICU), codes, and rapid response team (RRT) events [2-5]. However, few instances of these models have been shown to improve patient care, and there is little insight into how to implement interventions that use machine learning prediction models in the real-world setting [6]. A recently reported multisite prospective study of an intervention that used a model to predict inpatient clinical deterioration demonstrated improved clinical outcomes such as mortality rate and ICU length of stay in the intervention cohort, but there were no observed differences in the process measures, and it remains unclear which features of the implementation mediated the observed clinical benefit [7]. Therefore, although this particular intervention did demonstrate a clinical benefit at the participating study sites, there remains a pressing need for a deeper understanding of *how* such interventions can be effectively designed and implemented using AI to successfully disseminate them across other health care systems. As AI prediction capabilities in health care continue to grow, this implementation gap must be addressed to successfully leverage these capabilities to improve health care delivery.

One key question to address to close this implementation gap is how AI predictions can mediate changes within a complex work system to improve outcomes. Using a systems engineering lens, health care delivery—for example, for patients who are clinically deteriorating—can be viewed as occurring over a set of interconnected units (ie, people, technologies, and physical objects) that form distinct structures, processes, and patterns of behavior that lead to outcomes [8,9]. These health care work systems are typically considered to be *complex*, where the individual units dynamically interact with each other, self-organize, and adapt to the environment to form collective emergent properties that are difficult to predict and usually observed only after the system is live in the real world [10]. These systems are also described as *sociotechnical* because of the ways in which people and organizations (eg, patients and health care providers) interact with technology to make decisions, complete tasks, and form relationships and other organizational structures [11]. Emergent properties such as new workflows and habits, communication patterns, team structures, and cultures that arise from the introduction of new technologies may lead to unanticipated outcomes, barriers, or facilitators to the implementation of these technologies. In a systems thinking framework, the AI prediction model is not thought of as a standalone intervention but rather as an enabling component of a complex work system that mediates change [6].

Efforts to implement new technologies such as AI in health care often fail to consider the complexity of the sociotechnical systems in which these technologies are required to operate and underappreciate the unanticipated system-level effects that the technology may have on the health care delivery environment, and these effects can affect the success of the implementation [12]. For example, existing implementations of clinical deterioration prediction models typically assume a linear causal chain between the generation of an alert from the prediction model and the downstream actions of the receiving clinician who mediates the change in the outcome. However, they often do not explicitly consider and evaluate system-level properties such as the communication patterns and relationships among different members of the clinical team (eg, physicians and nurses), the degree to which the team members accurately share a mental model of risk, and the workflows and habits that evolve from the introduction of the AI prediction model. Evaluations of these implementations are therefore limited to only assessments of the clinical outcomes and select process metrics, and they do not capture the emergent properties that may help explain the barriers and facilitators to implementation and how the intervention mediates the changes in outcomes.

### Objectives

This paper presents a theory-driven study protocol informed by systems thinking and implementation science to assess the clinical and implementation outcomes of an implementation of a sociotechnical work system enabled by an AI prediction model for the early identification of hospitalized patients at risk of clinical deterioration. This protocol has three aims to (1) assess whether the AI-enabled work system improves clinical outcomes, (2) describe how the clinical deterioration predictive model and associated work processes are implemented, and (3) define the emergent properties of the AI-enabled work system that mediate the observed clinical outcomes.

## Methods

### Theoretical Framework

The Systems Engineering Initiative for Patient Safety (SEIPS) 2.0 model will be used as an analytic framework for this study [13]. The SEIPS model takes a person-centered approach to understand work processes within a sociotechnical system that is well suited to studying the impact of a technical solution (predictive model in the electronic health record [EHR]) on human work processes (management of hospitalized patients). The SEIPS framework characterizes outcomes as a product of a work system made up of factors related to the internal and external environment, people, organization, tools and technology, tasks, and work processes [8]. The revised model (SEIPS 2.0) incorporates the concepts of configuration, engagement, and adaptation, which better reflect a more dynamic implementation process among people, their environments, and the outcomes they produce [13].

### Setting

The intervention will be implemented in 6 primary general medicine adjustable acuity units at a quaternary academic hospital in the United States. The hospital has 605 beds, and most general medicine patients are cared for in 6 primary general medicine units. Each unit is served by physician teams, each with an average census of 12 and a maximum census of 20. The physicians spend most of their day rounding in many units and access the EHR through mobile devices, whereas the nurses spend most of their day in 1 unit working on a mobile desktop computer. These work setting characteristics were a key consideration in the intervention, implementation strategy, and study design.

### Intervention Description and Model Validation

The intervention is conceptualized as a work system comprising the following three parts: an AI model that predicts clinical deterioration, a mechanism for delivering the model predictions to clinical teams, and a multidisciplinary workflow driven by physicians and nurses. The clinical deterioration prediction model, the clinical deterioration index (CDI), was developed by Epic Systems Corporation and built into its EHR platform. The CDI is a logistic regression that runs every 15 minutes for all hospitalized patients using the most recent available clinical data of 31 physiological measures captured in the EHR and generates a score between 0 and 100, with higher scores indicating an increased risk of clinical deterioration as defined by any one of the following: ICU transfer, inpatient code, RRT event, or death. Between January 2020 and May 2020, the model was prospectively validated on 6232 hospital encounters of patients admitted to the implementation site for its accuracy in predicting ICU transfer or RRT event within 6-18 hours of the prediction, which was deemed by the clinical stakeholders to be an appropriate time interval that would allow for a meaningful clinical response. Of these 6232 encounters, 152 (2.44%) were unplanned ICU transfers and RRT events. The area under the curve was 0.70, and a model score threshold of 65 (out of 100) was chosen to maximize the positive predictive value and sensitivity of 0.20. Of note, this validation strategy was an enhancement of the vendor’s validation, which reported model accuracy in predicting the outcomes *without* the 6-hour to 18-hour time lag; this was thought to not be clinically meaningful because a model predicting an event to occur within the next 6 hours would not provide sufficient time for a clinical response to be effective.

Participatory design sessions using design thinking and process improvement methods were conducted with clinicians, including physicians, residents, and nurses. The participating clinicians generally preferred to be alerted only when patients were identified by the model as *high risk* rather than see the model prediction for every patient. Therefore, we designed the system to only flag patients whose CDI score was higher than 65, which, based on our validation, was the optimal cutoff to identify patients at the highest risk for unplanned ICU transfers and RRT events. As the area under the curve was only 0.70, the discriminatory ability of the model was not sufficiently robust to warrant showing the individual integer score values (ie, we did not want users to mistakenly interpret a higher integer score within the high-risk group as translating into higher risk) [2,4]. Rather, we incorporated a population-level description of patients in the high-risk group with the statement that the flagged patients have a *1 in 5 chance of requiring a RRT or ICU transfer within the next 6-18 hours,* which is derived from our validation.

Three preferred alerting mechanisms for a patient identified by the model as high risk were identified: alert mode 1: a noninterruptive flag icon that appears on the screen next to patients classified as high risk in the EHR patient list view, which allows clinicians to quickly see the names of all patients classified as high risk on one screen; alert mode 2: a banner visible on top of the screen once the chart of a flagged patient is opened that provides additional information about what *high risk* means, the accuracy of the CDI model, and specific next steps that should be taken to assess the risk of deterioration; and alert mode 3: an interruptive alert delivered to mobile devices that includes the same information as the banner at the time a patient first crosses the high-risk CDI threshold.

**Figure 1:**
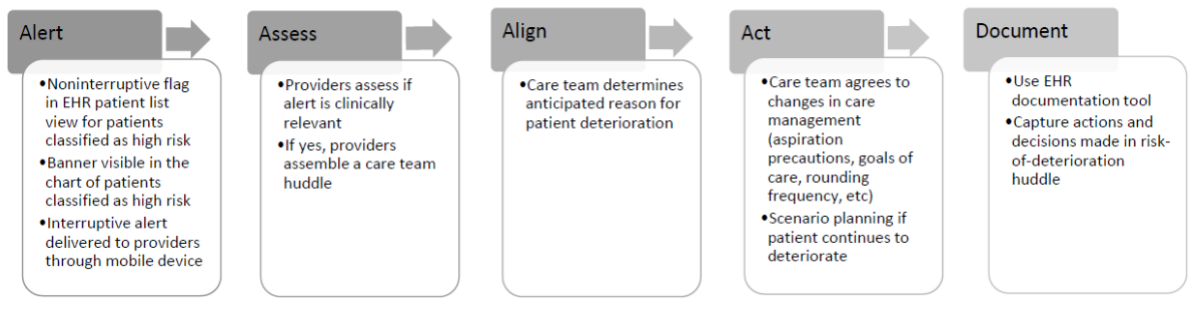
Workflow for assessing and managing clinical deterioration following an alert from the Clinical Deterioration Index prediction model. EHR: electronic health record.

Downstream workflows were designed to accompany these alert modes to improve the reliability and degree of team coordination in the clinical response for patients at risk of deterioration (Figure 1). Once the clinician receives the alert, they first assess the patient to judge whether the alert is clinically relevant and accurate and then conduct a huddle with the rest of the clinical team and use the following checklist to assess the patient’s risk and review mitigation strategies:

1. Align on the anticipated reason for patient deterioration.
2. Assess vital signs, airway or oxygenation needs, intravenous access, and code status.
3. Agree on changes to care management (eg, aspiration precautions in place, critical care consultation, etc).
4. Agree on the steps to take if the patient continues to deteriorate.

The last step involves documentation using an EHR-based documentation tool that captures the actions to be taken and the decisions made during this risk-of-deterioration huddle.

### Implementation Strategy

**Figure 2:**
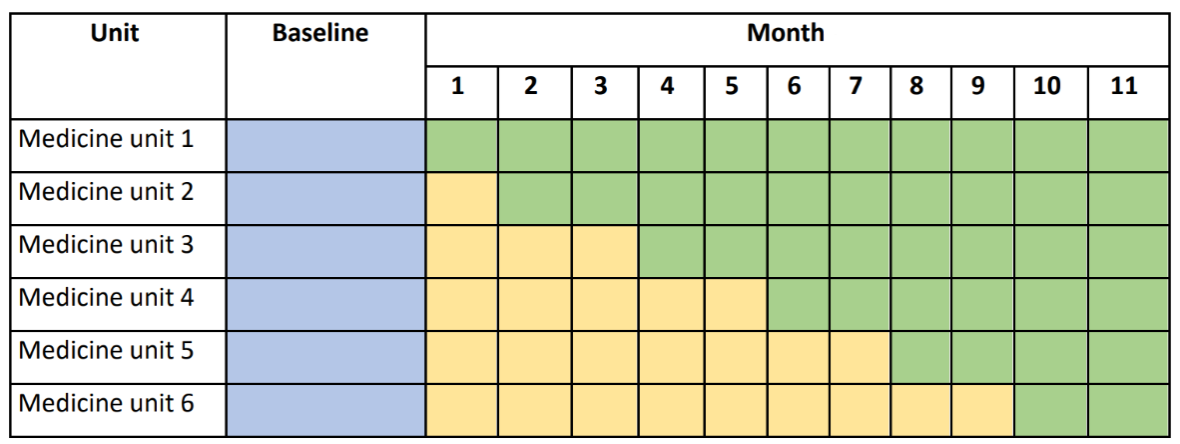
Stepped wedge design for the implementation and evaluation of a clinical deterioration model and workflow. Under *Baseline* column, blue shading includes data from the 10 months prior to the roll-out of the intervention for all 6 participating units (60-unit months for analysis). Under *Month* column, yellow shading represents stage 1 and the physician-only intervention for the physician-nurse team (25-unit months for analysis). Under *Month* column, green shading indicates stage 2 and the intervention for the physician-nurse team (41-unit months for analysis).

The intervention implementation will be split into two stages to assess its impact when offered to the physicians only (stage 1) and later to the entire physician-nurse team (stage 2). In stage 1 (yellow shading in Figure 2), the three alert modes will be delivered only to the physicians for patients across all 6 patient care units, and subsequent workflows will be initiated only by the physician.

In stage 2 (green shading in Figure 2), the alert modes will also be delivered to the nurses on the clinical teams, and either the physician or the nurse on the team can initiate the downstream workflow, thus creating a team approach to identifying and managing clinical deterioration. Nurses are to perform a parallel clinical validation of the alert and contact the physician to conduct the aforementioned risk-of-deterioration huddle. Thus, assessment of the risk and initial steps to manage deterioration can be initiated by either the physician or the nurses in the team intervention model. Stage 2 will be rolled out in a stepwise manner to each of the 6 primary general medicine units.

Rolling out the intervention using a stepwise approach was chosen for pragmatic implementation and evaluation purposes. The physicians care for patients across all 6 patient care units, whereas the nurses are staffed based on individual units. For implementation practicality and patient safety, stage 1 (physician-only intervention) would thus be implemented across all 6 units, whereas the implementation of stage 2 (the addition of nurses) would be staggered by the patient care unit so that the nurses in each unit can be trained in the new workflow together. This implementation strategy also allows us to evaluate the additive impact of delivering AI predictions to the entire physician-nurse team using a stepped wedge design.

### Study Design

To evaluate this complex, multicomponent intervention, we will use a mixed methods approach informed by the SEIPS 2.0 model to achieve our aims of assessing both the clinical outcomes and the processes that produced these outcomes. We found this model to be uniquely equipped to assess the impact of a new technology such as a predictive model because it takes into account both the social and technical aspects of implementation. Using a mixed methods approach will provide complementary data on the effect of the AI-enabled model on the management of clinical deterioration (ie, quantitative data will evaluate the clinical outcomes, and qualitative data will evaluate the processes) [14]. The intervention will be implemented consecutively in the medicine units based on a modified stepped wedge design featuring three stages (Figure 2): the baseline period (stage 0, blue shading) includes the 10-month period before the implementation of the intervention; in stage 1 (yellow shading), the intervention will be launched to all general medicine physicians across the hospital; and in the third and final phase (green shading), the intervention will be launched to the full physician-nurse clinical team in a staggered approach by medical unit. The study began in March 2021 (month 1).

### Sample

To evaluate this multidisciplinary intervention, data derived from multiple stakeholders, including providers, staff, and adult patients, will be included. The intervention will be implemented by providers and clinical staff in the 6 medicine units for all adult patients, as outlined in Figure 2. The clinical and process outcomes derived from the EHR and chart reviews will be captured at either the patient or unit level. The following patient inclusion criteria are based on the level of analysis:

- For unit-level outcomes, all patients admitted to the units will be included in the analyses.
- For patient-level outcomes, patients who cross the high-risk CDI threshold (CDI≥65) during their inpatient stay and will thus be eligible for the intervention will be included in the analyses.

For the qualitative evaluation, a stratified purposeful sample of approximately 20 providers and clinical staff who are typically responsible for identifying deterioration in patients will be selected for interviews (eg, hospital residents or interns, bedside nurses, critical care response team, clinical nurse specialists, and ICU fellows) [15].

### Outcomes

The clinical outcomes will be derived from data extracted from the patients’ EHRs related to inpatient hospital stay and up to 30 days after discharge to capture readmission. Using these data, the outcomes will be calculated and expressed at either the unit or patient level, as described herein. To facilitate generalizability and comparisons with other outcomes, the following outcomes, which are commonly assessed in other interventions for clinical deterioration [2,5,16], will be evaluated to address aim 1:

- Number of RRT activations in response to adverse events per unit per month (RRT/unit/month; unit level)
- Number of code events per unit per month (codes/unit/month; unit level)
- Number of ICU escalations per unit per month (ICU/unit/month; unit level)
- Mortality per unit per month (deaths/unit/month; unit level)
- Readmission rates (30-day rates) per unit per month (30-day readmissions/unit/month; unit level)
- Length of stay in acute inpatient setting (patient level nested in unit)

We will also explore the process outcomes informed by the SEIPS 2.0 model and the implementation outcomes described by Proctor et al [17] to assess the ways in which the model and workflow changed the work system and, subsequently, the work processes (aims 2 and 3). Where possible, these outcomes will be derived from the patients’ EHRs and a secure text messaging platform to minimize the required data reporting by clinicians. A focused chart review of a subsample of patients will also be conducted for data not extracted through the methods described previously. Examples of these processes include the following:

- Completion of documentation in the EHR (unit/month; unit level)
- Number and type of staff engaged in the intervention workflow (patient level nested in unit)
- Actions taken by the team engaged in the intervention workflow (patient level nested in unit)
- Time elapsed between alert and completion of workflow procedures, including documentation (patient level nested in unit)

### Data Collection and Procedures

Quantitative and qualitative data collection will be aligned to the SEIPS 2.0 framework. Mapping data to the framework aims to identify where multiple methods and sources of data can be used to explore each concept [14].

#### EHR Data Extraction

Quantitative data for processes and clinical outcomes will be extracted from the patients’ EHRs (Epic Systems Corporation) and the records of the clinical teams’ secure text messaging service (Voalte). Data extracted at the patient level will include notes, flowsheets of orders and referrals, and laboratory results, along with associated time stamps to assess the processes of the clinical teams’ response to clinical deterioration. Collecting these outcomes will require the development of electronic tools that will permit the capture and storage of these data discretely in the EHR as opposed to encounter-note text, for example.

#### Chart Review

Nondiscrete exploratory outcomes captured in the encounter notes will be investigated through chart review. To further explore the clinical processes related to the interruptive alert and clinical workflow, 10% of the patient charts will be reviewed to describe the workflow within the EHR after an alert has been triggered. The charts will be identified from a list of all patients for whom a high-risk alert was triggered during the evaluation period and stratified by unit to explore the potential between-units variation. Data elements and a template for the chart review will be determined based on the outlined SEIPS 2.0 constructs and will capture the events and orders documented in the EHR related to the CDI trigger, the location of documentation within the EHR, the role of the documenter, and interactions between the clinical team members. The chart review will follow the guidance described by Vassar and Holzmann [18].

#### Qualitative Interviews

Interviews with the clinicians will be conducted before and after the model and workflow are deployed to determine if and how the work system has changed (aims 2 and 3). The topic guide will be aligned with the concepts in the SEIPS 2.0 framework and the implementation outcomes [13,17]. The interviews are expected to take approximately 30-45 minutes and will be conducted by phone or video conference for the convenience of busy clinicians. The interviews will be recorded and transcribed for analysis. In addition, the outputs from the process improvement methods for designing the nursing workflow (process maps and pain point analysis) will be included as documentary data as part of the qualitative data set.

### Analysis

#### Quantitative Analysis

The primary aim is to assess the impact of the physician-only and team interventions on the unit-level clinical outcomes compared with baseline data. The secondary aim is to explore how the intervention is implemented by describing the process outcomes that were developed and launched as part of the intervention-specific workflow. For these process outcomes, the differences between the physician-only and team interventions will be statistically evaluated because baseline data will not be available for these outcomes. Poisson regression will be applied for unit-level count outcomes to determine the differences among the three phases of the study (baseline, physician-only, and team), followed by pairwise comparison. A multilevel, mixed-effects model will be used for patient-level outcomes to include the covariate unit to account for nesting. The level of statistical significance will be set at *P*<.05.

#### Power Calculation

**Figure 3:**
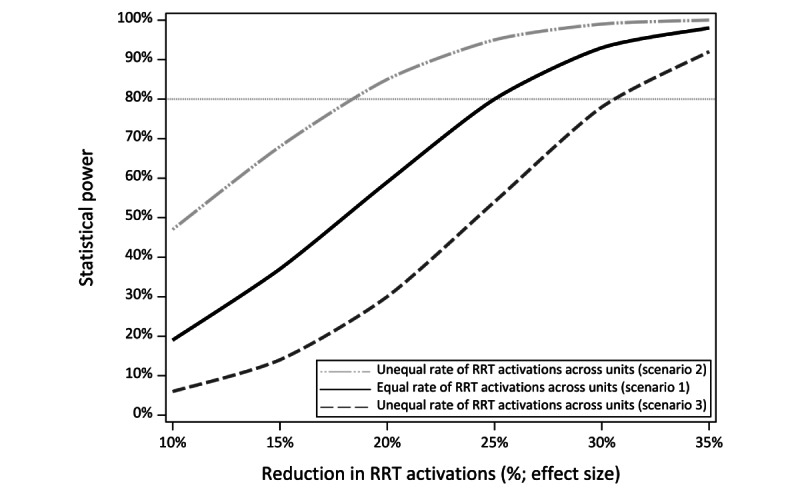
Power curve for simulations with both equal and unequal rapid response team rates across units. RRT: rapid response team.

On the basis of the aforementioned study design, we assessed the minimum effect size of the team intervention that can be detected with sufficient power (>80%). The percentage reduction in the number of RRT activations in response to adverse events per unit per month (RRT/unit/month) was chosen as the effect size measure because it is the most proximal of the clinical outcomes to the implemented workflow. As shown in Figure 2, our design provides a baseline period of 60 unit months and a team intervention period of 41 unit months. Assuming that the occurrence of RRT events follows a Poisson process, we conducted simulation studies with 10,000 replications to estimate the power curves using the following three scenarios. The first scenario assumes a mean rate of 4.5 RRT/unit/month during the baseline period for all 6 units. The second and third scenarios assume the following heterogeneous mean rates across the 6 units: 3.5 RRT/unit/month for 2 units, 4.5 RRT/unit/month for 2 units, and 5.5 RRT/unit/month for 2 units. In scenario 2, we assume that the team intervention is introduced to the units with lower rates at baseline first and the higher-rate units last, whereas in scenario 3, we assume the opposite. All scenarios use a two-sided α set at .05. Figure 3 shows the power curves for each scenario: scenarios 1, 2, and 3 indicate 80% power to detect a reduction in the RRT event rate of approximately 25%, 18%, and 30%, respectively.

#### Qualitative Analysis

A SEIPS 2.0 codebook will be created to define each of the concepts and create a shared mental model of the framework for clinical deterioration. Using the documentary data outputs from the process improvement methods to develop the workflow (process maps and pain point analysis) as a starting point, we will develop SEIPS configuration maps that determine important factors for identifying clinical deterioration related to task, person, organization, internal and external environments, and tools and technology—as well as their interactions. The members of the process improvement and evaluation teams will first meet to discuss the SEIPS codebook and then individually produce SEIPS 2.0 configuration maps. These maps will then be consolidated into one agreed configuration map that represents the current state of work for managing clinical deterioration. The SEIPS 2.0 configuration map will then be further refined with data from the interviews. NVivo (QSR International) will be used for data management and analysis. The interview analysis will use a deductive and inductive approach, using deductive codes derived from the SEIPS 2.0 framework and implementation outcomes while searching for emergent themes. The transcripts will be coded by one researcher with a portion of the transcripts coded by a second researcher to check for consistency in identifying themes [19]. The output from the qualitative analysis will be configuration maps before and after the implementation of the model and an assessment of the implementation outcomes, barriers, and facilitators.

#### Mixed Methods Analysis

Data will be mixed during the analysis phase using a mixed methods matrix [20]. Data collected using the aforementioned methods will be mapped to the SEIPS 2.0 framework (eg, data related to *tools and technology* will be gathered in the EHR by extracting number and pattern in the CDI views and in the interviews by asking questions about how the clinicians incorporate the CDI into their workflow and decision-making).

## Results

This study was given a nonresearch determination by the Stanford University Institutional Review Board because the purpose of this study is to develop and evaluate a system to identify and manage clinical deterioration that is specific to the hospital setting. The study began a pilot period of the intervention in one unit in December 2020, and the stepped wedge study began in March 2021.

## Discussion

This protocol paper proposes an approach to evaluation that recognizes the importance of assessing both processes and clinical outcomes to understand a multifaceted AI-enabled intervention aimed at the challenging problem of clinical deterioration. Our study design examines both clinical effectiveness and implementation outcomes because implementation outcomes such as clinician adoption, process metrics that reflect the impact of the machine learning model on workflow, and the clinical outcome of interest (eg, number of unplanned ICU transfers) are all intricately related as part of the work system. Evaluating these outcomes in tandem will help to understand how individual and team work processes mediate (or perhaps explain the lack of) any improvement in the clinical outcome, as well as any implications that the implementation may have for patient safety in the real-world setting. The implementation of this intervention reflects the real-world capabilities of currently available clinical deterioration prediction models, which have relatively low positive predictive value, and extends the question beyond the capabilities of the actual model to how AI models can inform, and be integrated into, the workflow. A complex systems approach using the SEIPS 2.0 framework to view the problem and the intervention will allow a more nuanced understanding of how a complex intervention involving AI and human behavior change interact to produce outcomes.

Traditional physician-nurse teams often face problems regarding hierarchy, communication breakdowns, and a lack of shared mental models for patient needs that result in challenges revolving around the management of high-stakes situations, such as clinical deterioration. Our evaluation will specifically assess if and how AI predictions, when incorporated into a team-based workflow, may help to reduce these barriers to effective team performance. This approach is a significant addition to the existing evaluations of AI interventions that primarily focus only on the effect of AI on clinical outcomes and do not sufficiently examine how the observed effects are mediated by AI and the associated work processes. Furthermore, the application of complexity and sociotechnical systems thinking in our evaluation allows for the generation of insights into how AI can be effectively incorporated into human work systems to deliver the desired improvements in processes and outcomes. The potential of AI to change the behavior of clinical teams is of particular interest to this evaluation.

The stepped wedge design demonstrates that limitations due to implementation requirements can be used as an opportunity to collect data to better differentiate the active ingredients (physician-only vs team) of a complex intervention. Our mixed methods approach aims to reflect the complexity of the system in which the AI-enabled CDI is deployed and provide detailed evidence of what works and why, thus providing the necessary foundation to ultimately support sustainability. Although we perceive that the team intervention will likely be specific to the hospital culture and setting in which it is implemented, using the SEIPS model as a theoretical approach to understand the impact on the work system will be of interest to a widespread audience looking to integrate AI into real-world clinical environments.

## Abbreviations

AI: artificial intelligence
CDI: clinical deterioration index
EHR: electronic health record
ICU: intensive care unit
RRT: rapid response team
SEIPS: Systems Engineering Initiative for Patient Safety

## References

[1] Ferreira FL, Bota DP, Bross A, Mélot C, Vincent JL (2001). Serial evaluation of the SOFA score to predict outcome in critically ill patients. J Am Med Assoc.

[2] Churpek MM, Yuen TC, Edelson DP (2013). Predicting clinical deterioration in the hospital: the impact of outcome selection. Resuscitation.

[3] Escobar GJ, Dellinger RP (2016). Early detection, prevention, and mitigation of critical illness outside intensive care settings. J Hosp Med.

[4] Lee J, Jung Y, Kim HJ, Koh Y, Lim C, Hong S, Huh JW (2020). Derivation and validation of modified early warning score plus SpO2/FiO2 score for predicting acute deterioration of patients with hematological malignancies. Korean J Intern Med.

[5] Loreto M, Lisboa T, Moreira VP (2020). Early prediction of ICU readmissions using classification algorithms. Comput Biol Med.

[6] Li RC, Asch SM, Shah NH (2020). Developing a delivery science for artificial intelligence in healthcare. NPJ Digit Med.

[7] Escobar GJ, Liu VX, Schuler A, Lawson B, Greene JD, Kipnis P (2020). Automated identification of adults at risk for in-hospital clinical deterioration. N Engl J Med.

[8] Carayon P, Schoofs HA, Karsh B, Gurses AP, Alvarado CJ, Smith M, Flatley BP (2006). Work system design for patient safety: the SEIPS model. Qual Saf Health Care.

[9] Holmes B, Finegood DT, Riley B, Brownson RC, Colditz GA, Proctor EA (2012). Systems thinking in dissemination and implementation research. Dissemination and Implementation Research in Health: Translating Science to Practice.

[10] Ratnapalan S, Lang D (2020). Health care organizations as complex adaptive systems. Health Care Manag (Frederick).

[11] Oosthuizen R, Pretorius L (2016). Assessing the impact of new technology on complex sociotechnical systems. S Afr J Ind Eng.

[12] Beede E, Baylor E, Hersch F, Iurchenko A, Wilcox L, Ruamviboonsuk P, Vardoulakis LM (2020). A human-centered evaluation of a deep learning system deployed in clinics for the detection of diabetic retinopathy. Proceedings of the 2020 CHI Conference on Human Factors in Computing Systems.

[13] Holden RJ, Carayon P, Gurses AP, Hoonakker P, Hundt AS, Ozok AA, Rivera-Rodriguez AJ (2013). SEIPS 2.0: a human factors framework for studying and improving the work of healthcare professionals and patients. Ergonomics.

[14] Palinkas LA, Aarons GA, Horwitz S, Chamberlain P, Hurlburt M, Landsverk J (2011). Mixed method designs in implementation research. Adm Policy Ment Health.

[15] Palinkas LA, Horwitz SM, Green CA, Wisdom JP, Duan N, Hoagwood K (2015). Purposeful sampling for qualitative data collection and analysis in mixed method implementation research. Adm Policy Ment Health.

[16] Kollef MH, Chen Y, Heard K, LaRossa GN, Lu C, Martin NR, Martin N, Micek ST, Bailey T (2014). A randomized trial of real-time automated clinical deterioration alerts sent to a rapid response team. J Hosp Med.

[17] Proctor E, Silmere H, Raghavan R, Hovmand P, Aarons G, Bunger A, Griffey R, Hensley M (2011). Outcomes for implementation research: conceptual distinctions, measurement challenges, and research agenda. Adm Policy Ment Health.

[18] Vassar M, Holzmann M (2013). The retrospective chart review: important methodological considerations. J Educ Eval Health Prof.

[19] Miles M, Huberman A, Saldana J (2013). Qualitative Data Analysis, 4th Ed.

[20] O'Cathain A, Murphy E, Nicholl J (2010). Three techniques for integrating data in mixed methods studies. Br Med J.

